# Comparison of clinical symptoms, gastric motility and fat intake in the early chronic pancreatitis patients with anti-acid therapy-resistant functional dyspepsia patients

**DOI:** 10.1371/journal.pone.0205165

**Published:** 2018-11-07

**Authors:** Mako Wakabayashi, Seiji Futagami, Hiroshi Yamawaki, Atsushi Tatsuguchi, Keiko Kaneko, Shuhei Agawa, Kazutoshi Higuchi, Noriko Sakasegawa, Makoto Murakami, Teppei Akimoto, Yasuhiro Kodaka, Nobue Ueki, Kaya Gudis, Chiaki Kawamoto, Takashi Akamizu, Choitsu Sakamoto, Katsuhiko Iwakiri

**Affiliations:** 1 Department of Internal Medicine, Division of Gastroenterology, Nippon Medical School, Tokyo, Japan; 2 The First Department of Medicine, Wakayama Medical Unversity, Wakayama, Japan; University Hospital Llandough, UNITED KINGDOM

## Abstract

**Background:**

There was no available data concerning the clinical differentiation between the updated definition of early chronic pancreatitis (ECP) and anti-acid therapy-resistant functional dyspepsia (RFD).

**Aims:**

We aimed to determine whether clinical symptoms, gastric motility, psychogenic factors and fat intake can help distinguish early chronic pancreatitis (ECP) from anti-acid therapy-resistant functional dyspepsia patients with pancreatic enzyme abnormalities (RFD-P) and anti-acid therapy-resistant functional dyspepsia (RFD) patients using endosonography.

**Methods:**

We enrolled 102 consecutive patients presenting with typical symptoms of RFD patients (n = 52), ECP patients (n = 25) and RFD-P patients (n = 25). ECP patients were diagnosed based on the criteria recommended by the Japan Pancreatic Association. Gastric motility was evaluated by ^13^C-acetate breath tests. Severity of duodenal inflammation was examined.

**Results:**

24.5% of RFD patients were determined as ECP using endosonography. Abdominal pain score in Gastrointestinal Symptom Rating Scale (GSRS) in the patients with ECP was significantly lower compared to that in the patients with RFD-P. There were no significant differences in State-Trait Inventory (STAI)-state/-trait scores, Self-Rating Questionnaire for Depression (SRQ-D) scores and clinical symptoms for fat intake among three groups. The early phase of gastric emptying (AUC_5_; AUC_15_) in ECP and RFD-P patients were significantly disturbed compared to those in RFD patients.

**Conclusions:**

Evaluation of severity of abdominal pain and measurement of the early phase of gastric emptying will be useful tools to distinguish ECP patients from RFD patients. Accurate diagnosis of ECP patients may contribute to the prevention from advancing of chronic pancreatitis.

## Introduction

According to the Rome III classification criteria, the major symptoms of functional dyspepsia (FD) consist of bothersome postprandial fullness, early satiety, epigastralgia and epigastric burning [1, 2]. The symptom pattern and underlying pathology of FD are heterogeneous. Thus, visceral hypersensitivity in response to distention [3], impaired meal accommodation [4] and delayed gastric emptying have frequently been demonstrated in patients diagnosed with FD [5–7]. Since FD patients, especially those with epigastric pain syndrome (EPS), can be classified into heterogenic subgroups, the use of proton pump inhibitors in the treatment of EPS remains controversial [8–11]. Since FD patients were determined based on their bothersome symptoms in spite of no existence of organic diseases using abdominal ultrasonography or abdominal CT scanning, FD patients could involve chronic pancreatitis patients accompanying with abdominal fullness, epigastric pain and early satiety [12]. Anderson et al have reported that 35% of patients with dyspepsia suffer from pancreatic enzyme abnormalities [13]. In addition, 27% of subjects with functional dyspepsia have been reported to have pancreatic juice abnormalities consistent with chronic pancreatitis [14]. Ashizawa et al have also reported that anti-acid therapy-resistant FD (RFD) involves FD patients with concomitant with chronic pancreatitis [15]. Of interest, a high fat diet has been reported to aggravate symptoms of abdominal pain and abdominal fullness in FD patients [16, 17] as well as in patients with chronic pancreatitis [18]. Moreover, fat intake is the strongest nutritional stimulant of pancreatic secretion [19]. However, few studies have reported an association between a high fat diet and the risk of chronic pancreatitis or chronic pancreatitis related complications [18–20]. Thus, it is critical to clarify the etiology of these two distinct diseases to determine the association between high levels of fat intake and the aggravation of certain clinical symptoms in functional dyspepsia and chronic pancreatitis patients.

Sahai et al have also reported that dyspepsia may be an atypical presentation of pancreatic disease using endoscopic ultrasonography (EUS) [11]. In Japan, to hinder the initial phase of chronic pancreatitis from advancing into chronic pancreatitis, new strategies for addressing chronic pancreatitis in its early stages have been proposed [21]. According to the Japan Pancreatic Association (JPA), four clinical criteria including epigastric pain and the presence of more than two features of EUS are needed for a diagnosis of early chronic pancreatitis (ECP) [21]. In 2015, the Japanese society of Gastroenteology (JSGE) approved new guidelines for functional dyspepsia, which disclosed that 24% of FD cases involved chronic pancreatitis [13, 22]. However, there were no data available concerning the clinical differentiation between the updated definition of ECP and FD patients. In this study, we aimed to determine whether there were any differences in clinical symptoms, gastric motility, psychogenic factors and the effect of fat intake among patients diagnosed with ECP and FD patients refractory to anti-acid therapy with or without pancreatic enzyme abnormalities using endosonography.

## Methods

### Patients

**Fig 1:**
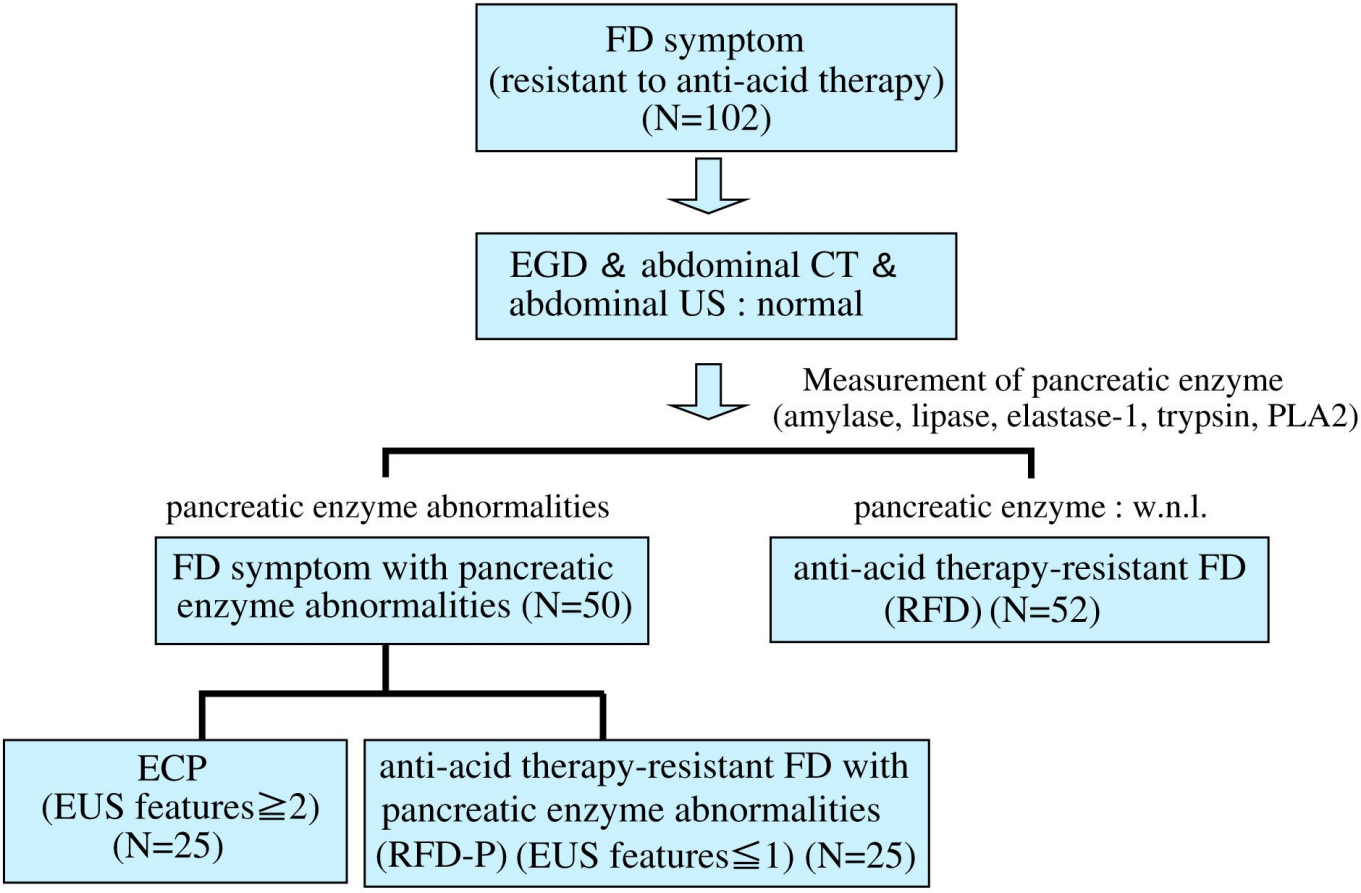
Flowchart of this study.

This study enrolled 102 consecutive patients presenting with anti-acid therapy-resistant FD patients without pancreatic enzyme abnormalities (RFD) (n = 52), early chronic pancreatitis (ECP) (n = 25) and anti-acid therapy-resistant FD patients with pancreatic enzyme abnormalities (RFD-P) (n = 25), after upper gastrointestinal endoscopy, abdominal ultrasonography and abdominal computed tomography from April 2013 to April 2016 (Fig 1). According to the lack of pancreatic enzyme abnormalities, we first determined RFD patients and later the others were divided with the EUS results in ECP and RFD-P patients (Fig 1). Patients were diagnosed according to the Rome III criteria [2]. Exclusion criteria included severe heart disease, renal or pulmonary failure, liver cirrhosis, severe systemic illness and history of malignant disease. Patients with previous gastroduodenal surgery, gastroduodenal ulcers, duodenal ulcer scars, severe duodenitis, biliary dyskinesia, diabetes mellitus and recent use of non-steroidal anti-inflammatory drugs (NSAIDs) or anticoagulants at endoscopy were also excluded. *Helicobacter pylori (H*. *pylori)* infection was determined by both the ^13^C-urea breath test and by measurement of anti-*H*. *pylori* antibody. We measured amylase, lipase, trypsin, PLA2 and elastase-1 in the sera of the RFD patients. Written informed consent was obtained from all subjects prior to undergoing upper gastrointestinal endoscopy and abdominal ultrasonography for evaluation of dyspeptic symptoms. The study protocol was approved by the Ethics Review Committee of Nippon Medical School Hospital.

### Clinical symptoms

Clinical symptoms of FD were evaluated according to the Rome III criteria [2]. Clinical symptoms must have involved at least one of the following: early satiation, bothersome postprandial fullness, epigastric pain and epigastric burning. Diagnosis for PDS and EPS was fulfilled with symptoms occurring for the last three months and the onset of symptoms occurring at least six months prior to diagnosis. In this study, FD symptoms including epigastric pain, epigastric burning, postprandial fullness and early satiety, and satisfaction with treatment, were assessed based on Rome III classification [2] and scored as follows: 0, none; 1, very mild; 2, mild; 3, moderate; 4, severe; and 5, very severe. RFD patients were determined when an 8-week proton pump inhibitor (PPI) treatment protocol failed to improve FD symptoms down to levels less than Score 2. Clinical symptoms were evaluated with the Gastrointestinal Symptom Rating Scale (GSRS) [23]. The GSRS is composed of 15 items that generate 5 components including gastroesophageal reflux, abdominal pain, indigestion, diarrhea and constipation. Each item was rated according to severity on a scale of 1 (no discomfort at all) to 7 (very severe discomfort). We used the mean score of the GSRS and the 15 GI symptoms of the GSRS to evaluate dyspeptic symptoms.

### Clinical symptoms for fat intake

Patients reported their own clinical symptoms after eating each of thirteen different foods (fried shrimp, processed cheese, croissants, meat balls, tomatoes, oranges, asparagus, hamburger, milk, apples, chicken nuggets, yogurt, and sweet potatoes) that included high-fat meals for a period of one month. In this study, high-fat meals were defined as those containing more than 16 grams of fat per 100 grams of food, such as fried shrimp (20.3g fat/100g), processed cheese (26g fat/100g), croissants (26.8g fat/100g) and meatballs (16.4g fat/100g) based on previous study [24]. Patients consuming high-fat meals evaluated their own clinical symptom scores as follows: 0, no complaints; and 1, presence of any clinical symptoms including gastroesophageal reflux, abdominal pain, dyspepsia, diarrhea and constipation.

### Assessment by endosonography

An Olympus EUS-UCT 260 convex scanning endosonography (Olympus America, Melville, NY) at 7.5 MHz was used to perform EUS under conscious sedation in 50 FD patients with pancreatic enzyme abnormalities (Fig 1). Endosonographic parenchymal or ductal abnormalities were recorded. These abnormalities were defined as follows: lobularity with honeycombing, lobularity without honeycombing, hyperechoic foci without shadowing stranding, cysts, dilated side branches and hyperechoic main pancreatic duct (MPD) margin [21]. When opinions differed among expert endoscopists, a final judgement was arrived at by consensus following a discussion of each individual case. Diagnosis of early chronic pancreatitis was made with imaging findings of 2 or more EUS features among the seven features listed above, and clinical findings of two or more symptoms including repeated attacks of upper abdominal pain, abnormalities in blood/urine pancreatic enzymes, exocrine pancreatic dysfunction and a history of chronic alcohol intake (80g/day) (Fig 1).

### Duodenal inflammatory cells infiltration

Histological duodenitis was assessed as mild, moderate, or severe by H&E staining according to criteria of previous studies [25]. Mild duodenitis was defined as an expansion of the lamina propria by mild inflammatory cell infiltration. Moderate duodenitis was characterized by partial loss of villi and expansion of the lamina propria by moderate inflammatory cell infiltration. Severe duodenitis was characterized by partial loss of villi and expansion of the lamina propria by severe inflammatory cell infiltration, mainly plasma cells, macrophages, and lymphocytes. Mild to severe duodenal inflammatory cell infiltration was evaluated by the degree (0–3) of mononuclear cell infiltration. Severity of inflammatory cells were classified as 0 = normal, 1 = mild; diffuse superficial infiltration, 2 = moderate; extending into the middle of the mucosa, 3 = severe; transmucosal infiltration according to the modified criteria of the previous report [26]. Specimens were evaluated by two experienced pathologists in a blinded manner.

### State-Trait Anxiety Inventory (STAI)

We evaluated anxiety using the Japanese version of the State-Trait Anxiety Inventory (Form X). The State-Trait Anxiety Inventory was developed by Spielberger et al [27] to determine individuals’ separate state and trait anxiety levels and has been standardized for Japan. The STAI is a well-validated 40-item self-reported questionnaire to evaluate degree of anxiety [27]. State of anxiety reflects a “transitory emotional state or condition of the human organism that is characterized by subjective, consciously perceived feelings of tension and apprehension, and heightened autonomic nervous system activity.” State of anxiety may fluctuate over time and can vary in intensity. In contrast, trait of anxiety denotes “relatively stable individual differences in anxiety proneness”.

### Self-Rating Questionnaire for Depression (SRQ-D)

Status of depression was evaluated by Self-Rating Questionnaire for Depression. The SRQ-D comprises 18 items, which are rated on a 4-point scale (0 = ‘no’, 1 = ‘sometimes’, 2 = ‘frequently’, and 3 = ‘always’). Among these 18 questions, 6 non-relevant questions are interspersed. According to the diagnostic criteria for depression, subjects with scores of 9 points or less are considered to be normal; those with scores of 10–15 points as borderline; while those with scores of 16 points or more, as having mild depression. In this study, we determined patients having SRQ-D scores of 16 points or more as having symptoms of depression [28].

### Pittsburgh Sleep Quality Index (PSQI)

Sleep quality and sleep duration were evaluated by a Japanese version of the Pittsburgh Sleep Quality Index (PSQI) questionnaire [29]. The score of each component ranges from 0 to 3, reflecting severity of symptoms, and the sum of the seven component scores provide a global PSQI score that ranges from 0 to 21. Higher scores indicate poorer sleep [29, 30]. A cut-off score >5.5 has a sensitivity of 80.0–85.7% for various patient groups, and a specificity of 86.6% for control subjects in the Japanese version of the PSQI [29].

### Measurement of gastric emptying

Sodium acetate (water soluble,^13^C-acetate) for emptying of liquids was used as a tracer (Cambridge Isotope Laboratories; Tewksbury, MA). The liquid test meal consisted of 100mg of ^13^C-acetate dissolved in 200 ml of a liquid meal (Racol, 1ml/1kcal; Otsuka Pharmacia Company, Tokyo, Japan). Enrolled patients were fasting in 12 hours for measurement of gastric emptying. Breath samples were collected 0 sec, 10 sec, 5 min, 10 min, 15 min, 20 min, 30 min, 40 min, 50 min, 60 min, 75 min and 90 min after ingestion of the test meal at 10:00 a.m. The subject’s own production of 300mmol CO_2_ per m^2^ body surface and per hour was set as default. We used an Integrated Software Solutions program to calculate the half gastric emptying time (T_1/2_) and the lag phase (Tmax; min) as the point of maximum gastric emptying according to Hellmig et al [31]. The half gastric emptying time (T_1/2_) represents the time when 50% of the initial gastric content was emptied. A Tmax value greater than 60 min, representing the mean Tmax in healthy volunteers plus SD, was defined to represent relative disturbances in gastric emptying according to the diagnostic criteria of the Japan Society of Smooth Muscle Research and our study [32, 33].

### Data analysis

The time plot of pulmonary [^13^CO_2_] excretion (%dose/hr) was fitted to the function: (% dose / hr) = m × k × β × e^−kt^ × (1 − e^−kt^)^β−1^ where “m” is the cumulative [^13^CO_2_] recovery at the infinite time, “t” is in hours and “k” and “β” are regression-estimated constants.

$$(Cumulative\%dose)=m\times {\left(1-e^{-kt}\right)}^{\beta }$$

$${AUC}_{5}=m\times {\left(1-e^{-k\times 0.08t}\right)}^{\beta }\;[T:\;5\;\min=0.08\;\mathrm{hr}]$$

$${AUC}_{15}=m\times {\left(1-e^{-k\times 0.25t}\right)}^{\beta }\;[T:\;15\;\min=0.25\;\mathrm{hr}]$$

$${AUC}_{30}=m\times {\left(1-e^{-k\times 0.5t}\right)}^{\beta }\;[T:\;30\;\min=0.5\;\mathrm{hr}]$$

$${AUC}_{60}=m\times {\left(1-e^{-k\times 1.0t}\right)}^{\beta }\;[T:\;60\;\min=1.0\;\mathrm{hr}]$$

$${AUC}_{90}=m\times {\left(1-e^{-k\times 1.5t}\right)}^{\beta }\;[T:\;90\;\min=1.5\;\mathrm{hr}]\;(\mathrm{AUC}:\;\mathrm{area}\;\mathrm{under}\;\mathrm{the}\;\mathrm{curve})$$

We determined the area under the curve at 5-minute (AUC_5_) and 15-minute (AUC_15_) values as markers of the early phase of gastric emptying based on previous studies [34, 35]. AUC_5_ values >17.4 and AUC_15_ values >39.6, representing the mean AUC value of healthy volunteers plus 2SD, were defined to represent disturbances in the early phase of gastric emptying.

### Sample size

In our study, we determined the sample size using the PS (Power and Sample size calculations program) software program, a gift from Vanderbilt University. The standard deviation of the AUC_5_ value for the RFD patients was approximately 5.86 (σ = 5.86). Using the above data, setting α = 0.05, β = 0.80, and the estimated mean score of AUC_5_ in patients with ECP as 24.2; 25 ECP patients, 25 RFD-P patients and 52 RFD patients were estimated to be sufficient to identify clinically relevant differences. In addition, the standard deviation of the abdominal pain value for the RFD-P patients was approximately 1.19 (σ = 1.19). Using the above data, setting α = 0.05, β = 0.80, and the estimated mean score of abdominal pain in patients with ECP as 2.11; 25 ECP patients, 25 RFD-P patients and 52 RFD patients were also estimated to be sufficient to identify clinically relevant differences.

### Statistical analysis

For statistical evaluation of group data, Students’ t-test for paired data and analysis of variance (ANOVA) for multiple comparisons were followed by Scheffe’s F test. The Mann-Whitney U test was used for analysis of categorical data. Data analyses were performed by using a standard software package (SPSS version 13.0, Chicago, IL). A *P* value of less than 0.05 was statistically significant.

## Results

### Characteristics of ECP patients, RFD-P patients and RFD patients

Age, alcohol consumption, smoking rate, positivity of *H*. *pylori* infection and total GSRS score did not differ statistically among early chronic pancreatitis patients (ECP) (n = 25), anti-acid therapy-resistant FD patients with pancreatic enzyme abnormalities (RFD-P) (n = 25) and anti-acid therapy-resistant FD patients without pancreatic enzyme abnormalities (RFD) (n = 52) (Table 1). On the other hand, the proportion of female patients was significantly (P = 0.0031) higher in RFD-P patients than in RFD patients (Table 1). In addition, there were no significant differences in amylase, lipase, trypsin, elastase-1 or PLA2 between ECP and RFD-P patients. ECP was determined as the presence 2 or more EUS features; i.e., scores≥2.(Fig 1). In ECP patients, the percentages of score 2, score 3 and score 4 of EUS features were 64%, 28% and 8%, respectively (Table 1). In comparison, in RFD-P patients, the percentages of EUS features was 52% for score 0 and 48% for score 1 using endosonography (Table 1).

**Table 1:** Characteristics of ECP patients, RFD-P patients and RFD patients.

Factors		ECP (n = 25)	RFD-P (n = 25)	RFD (n = 52)
Age		59.5±3.06	58.5±3.18	58.5±2.59
Sex (F/M)		16/ 9	20/ 5 *	23/ 29
Total GSRS		2.27±0.11	2.73± 0.25	2.70±0.18
Positivity of H. pylori infection		8%	20%	9.62%
Alcoholconsumption (mL)		50.9±18.8	45±25.0	8.90±7.16
Smoking rate		28%	21%	29%
The score of EUS features	Score 0		52%	
	Score 1		48%	
	Score 2	64%		
	Score 3	28%		
	Score 4	8%		

ECP: Early chronic pancreatitis, RFD-P: Anti-acid therapy-resistant functional dyspepsia with pancreatic enzyme abnormalities, RFD: Anti-acid therapy-resistant functional dyspepsia, GSRS: Gastrointestinal Symptom Rating Scale RFD-P vs RFD

*p = 0.0031

### Comparison of clinical symptoms and bothersome FD symptoms among ECP patients, RFD-P patients and RFD patients

**Fig 2:**
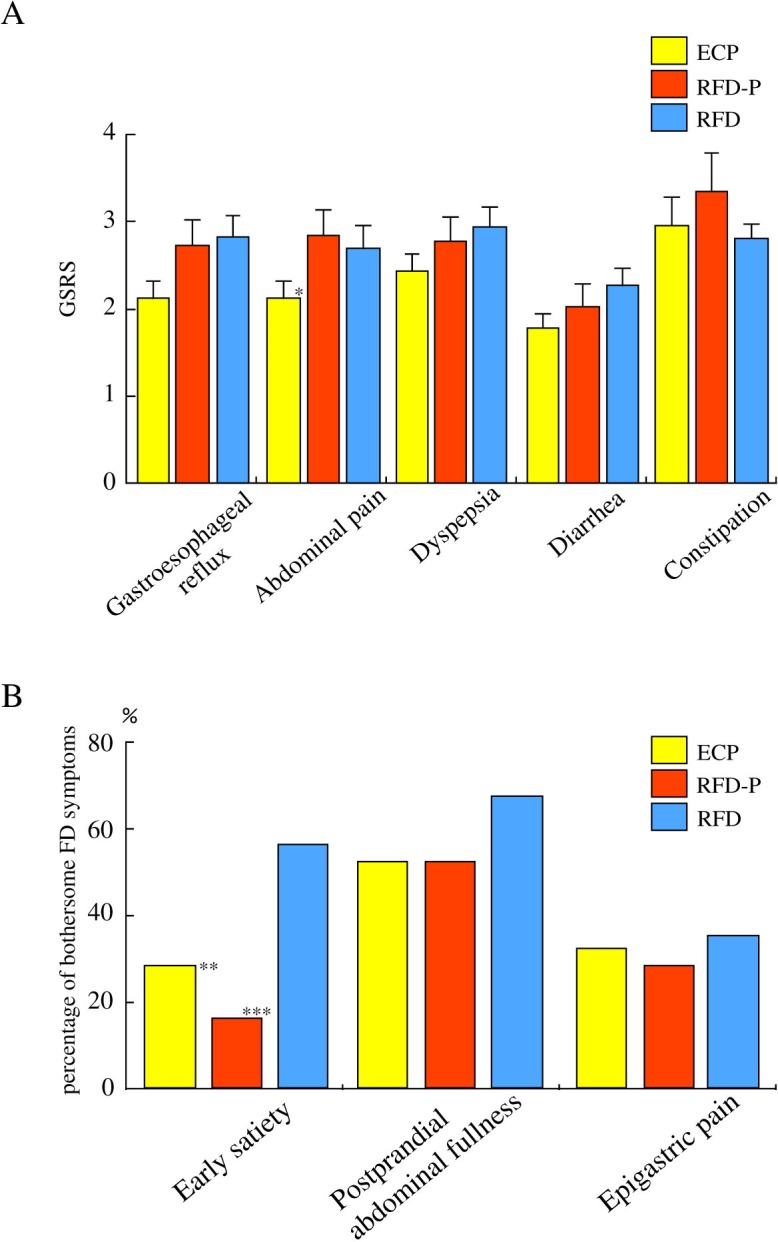
Comparison of clinical symptoms and bothersome FD symptoms among ECP patients, RFD-P patients and RFD patients. *A*. Comparison of clinical symptoms based on GSRS among the three groups.Abdominal pain score (2.11±0.21) in GSRS in the patients with ECP was significantly lower compared to that (2.83±0.30) in the patients with RFD-P. * P = 0.04, vs RFD-P patients. There were no significant differences in the grades of gastroesophageal reflux, dyspepsia, diarrhea and constipation among ECP, RFD-P and RFD patients.*B*. Comparison of bothersome FD symptoms among the three groups. There were significant differences in the percentages of severe early satiety (score>4) in ECP patients and RFD-P patients were significantly lower (P = 0.02 and P = 0.01, respectively) compared to that in RFD patients. **P = 0.02,vs RFD patients. ***P = 0.001, vs RFD patients.

To investigate whether there were any differences in clinical symptoms among ECP, RFD-P and RFD patients, we compared clinical symptoms based on GSRS scores among the three groups. Since abdominal pain was considered as one of the critical criteria for the diagnosis of ECP, we focused on the grade of abdominal pain in both ECP and RFD-P patients. Interestingly, abdominal pain score (2.11±0.21) in GSRS score in the patients with ECP was significantly (P = 0.04) lower compared to that (2.83±0.30) in the patients with RFD-P (Fig 2*A*). There were no significant differences in gastroesophageal reflux (2.11±0.21, 2.72±0.30, 2.81±0.25), dyspepsia (2.42±0.20, 2.77±0.27, 2.93±0.23), diarrhea (1.77±0.17, 2.02±0.26, 2.26±0.20) and constipation (2.94±0.34, 3.33±0.45, 2.80±0.16) among ECP, RFD-P and RFD patients (Fig 2*A*). In addition, we precisely focused on FD symptoms among the three groups. There was no significant difference (32% and 28%, respectively) in severity of epigastric pain between ECP and RFD-P patients (Fig 2*B*). Then, to investigate whether postprandial distress symptoms also contribute to the development of ECP patients, we evaluated postprandial distress symptoms such as postprandial abdominal fullness and early satiety among the three groups. There was no significant difference (52%, 52%; p>0.999) in the percentage of severe postprandial abdominal fullness (score >4) between ECP and RFD-P patients (Fig 2*B*). In addition, there was also no significant difference (28%, 16%; p = 0.06) in the percentage of severe early satiety (score >4) between ECP and RFD-P patients (Fig 2*B*).

Moreover, there were significant differences in the percentages of severe early satiety (score>4) in ECP patients (28%) and RFD-P patients (16%) were significantly lower (P = 0.02 and P = 0.001, respectively) compared to that in RFD patients (56%) (Fig 2B).

### Comparison of STAI, SRQ-D, and global PSQI scores among ECP patients, RFD-P patients and RFD patients

To compare psychogenic factors such as anxiety and depression among ECP, RFD-P and RFD patients, we estimated STAI-state/-trait and SRQ-D scores among the three groups. There were no significant differences in STAI-state/-trait and SRQ-D scores among the three groups (Table 2). There were also no significant differences in global PSQI scores among the three groups (Table 2).

**Table 2:** Comparison of STAI, SRQ-D and global PSQI scores among ECP patients, RFD-P patients and RFD patients.

Factors	ECP (n = 25)	RFD-P (n = 25)	RFD (n = 52)
STAI-state	50.7±6.95	50.0±6.17	46.9±4.11
STAI-trait	44.3±6.63	52.9±7.72	45.7±4.23
SRQ-D	10.1±1.01	13.1±1.31	11.2±0.78
Global-PSQI	5.53±0.57	6.19±0.22	6.67±0.51

STAI: State-Trait Anxiety Inventory, SRQ-D: Self-Rating Questionnaire for Depression, PSQI: Pittsburgh Sleep Quality Index, ECP: Early chronic pancreatitis, RFD-P: Anti-acid therapy-resistant functional dyspepsia patients with pancreatic enzyme abnormalities, RFD: Anti-acid therapy-resistant functional dyspepsia

### Comparison of gastric motility among ECP patients, RFD-P patients and RFD patients

To clarify whether there were significant differences in gastric emptying among ECP, RFD-P and RFD patients, we measured Tmax and T_1/2_ values such as gastric emptying, and AUC_5_ and AUC_15_ values such as the early phase of gastric emptying among the three distinct groups.

**Fig 3:**
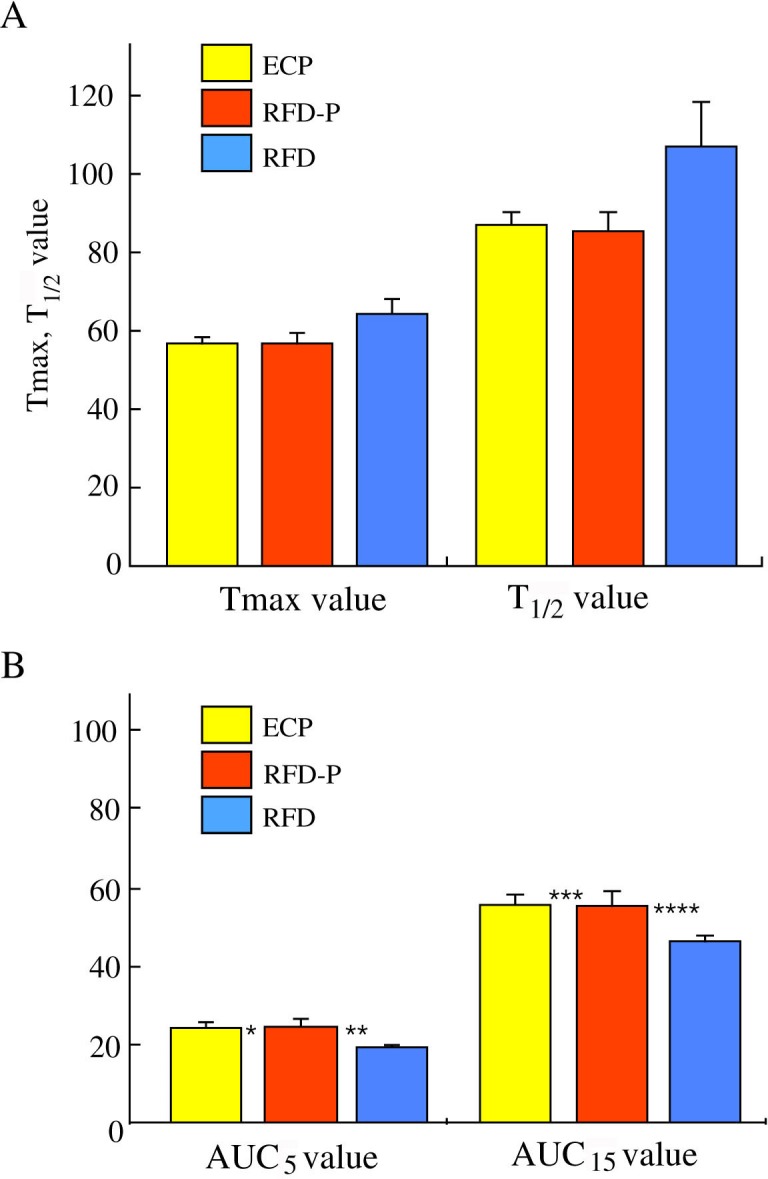
Comparison of gastric motility among ECP patients, RFD-P patients and RFD patients. *A*. There were no significant differences in Tmax and T_1/2_ values among early chronic pancreatitis (ECP), anti-acid therapy-resistant FD patients with pancreatic enzyme abnormalities (RFD-P) and anti-acid therapy-resistant FD (RFD) patients. *B*. AUC_5_ values (24.7±1.57 and 24.6±2.00, respectively) in ECP and RFD-P patients were significantly higher compared to that (19.2±0.81) in RFD patients. AUC_15_ values (55.8±2.73 and 55.4±3.94, respectively) in ECP and RFD-P patients were also significantly higher compared to that (46.3±1.57) in RFD patients. * P = 0.003, vs RFD patients, ** P = 0.004, vs RFD patients, *** P = 0.004, vs RFD patients, **** P = 0.001, vs RFD patients.

There were no significant differences in Tmax (56.6±1.75, 56.6±3.03 and 64.0±4.08, respectively) and T_1/2_ (87.0±3.26, 85.3±5.21, and 107±11.3, respectively) values among ECP, RFD-P and RFD patients (Fig 3*A*). Then, to determine whether the early phase of gastric emptying in ECP patients differs from that in RFD-P patients, we measured AUC_5_ and AUC_15_ values in each group. Interestingly, AUC_5_ values (24.7±1.57 and 24.6±2.00, respectively) in ECP and RFD-P patients were significantly (P = 0.003 and P = 0.004, respectively) higher compared to that (19.2±0.81) in RFD patients (Fig 3*B*). In addition, AUC_15_ values (55.8±2.73 and 55.4±3.94, respectively) in ECP and RFD-P patients were also significantly (P = 0.004 and P = 0.001, respectively) higher compared to that (46.3±1.57) in RFD patients (Fig 3*B*). However, there were no significant differences in AUC_5_ and AUC_15_ values between ECP and RFD-P patients (Fig 3*B*).

### Comparison of clinical symptoms for fat intake among each group

**Fig 4:**
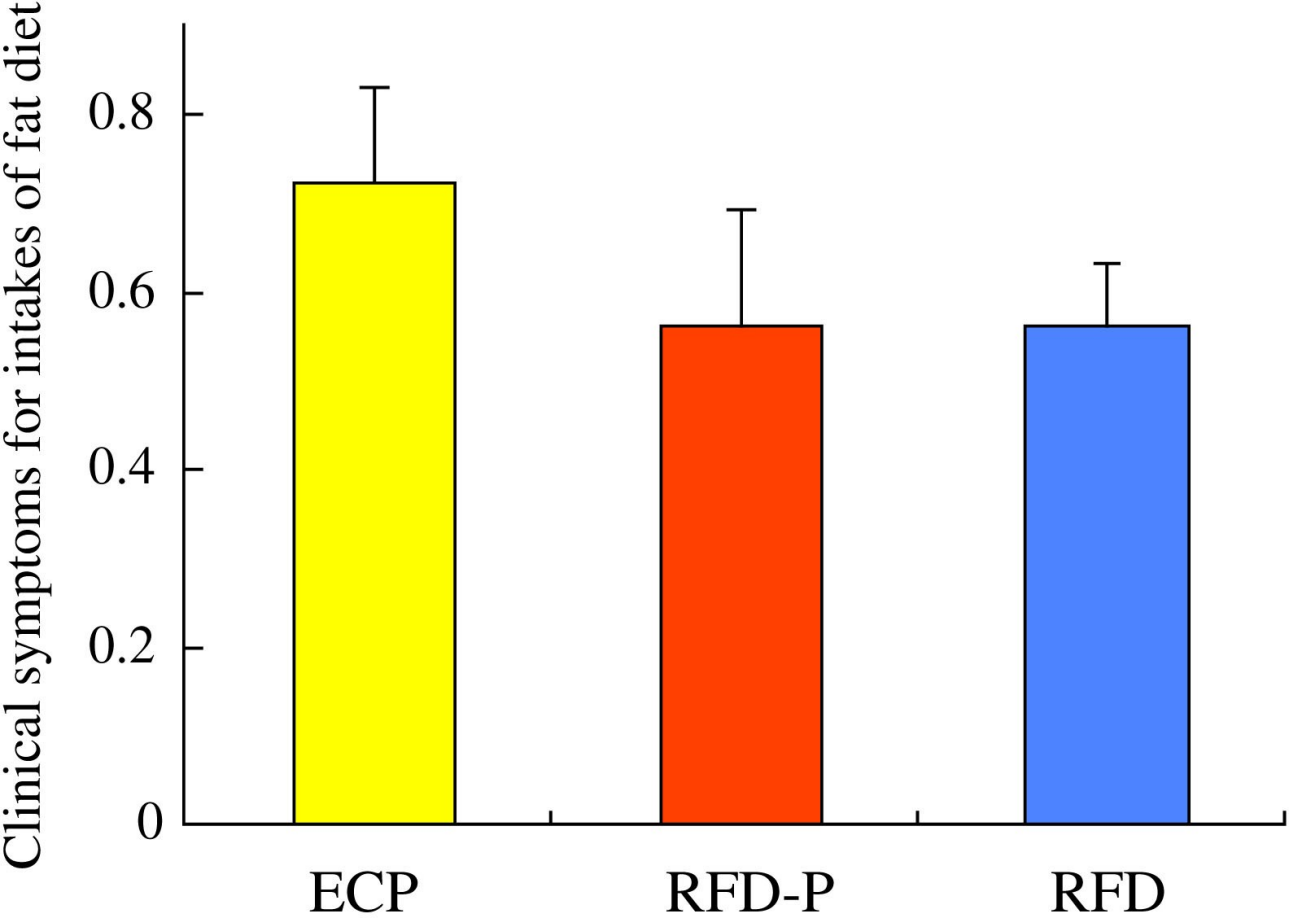
Comparison of clinical symptoms for fat intake among each group. Score of clinical symptoms (0.72±0.11) for fat intake in early chronic pancreatitis (ECP) patients did not significantly differ from those (0.56±0.13, 0.56±0.07) in anti-acid therapy-resistant FD patients with pancreatic enzyme abnormalities (RFD-P) and anti-acid therapy-resistant FD patients (RFD).

To investigate clinical symptoms for fat intake among each group, we estimated aggravation of clinical symptoms after fat intake among each group. Score of clinical symptoms (0.72±0.11) for fat intake in ECP patients did not significantly differ from those (0.56±0.13, 0.56±0.07) in RFD-P and RFD patients (Fig 4).

### Relationship between duodenal inflammatory cells infiltration and the early phase of gastric emptying in ECP patients and RFD-P patients

**Fig 5:**
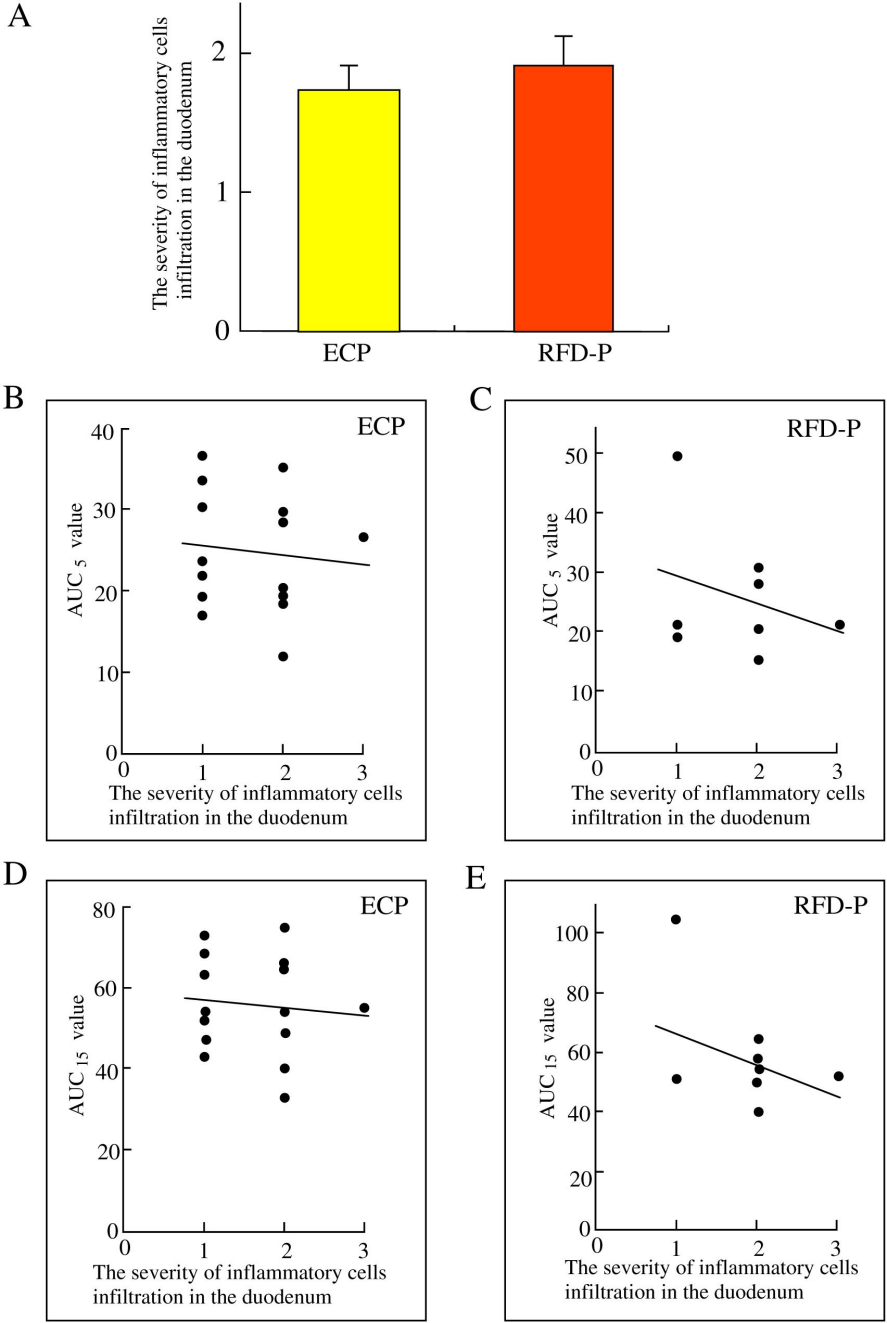
Relationship between duodenal inflammatory cell infiltration and early phase of gastric emptying in ECP patients and RFD-P patients. *A*. The severity of inflammatory cells infiltration in the duodenum of the early chronic pancreatitis (ECP) patients and anti-acid therapy-resistant FD patients with pancreatic enzyme abnormalities (RFD-P). *B*. The relationship between the severity of inflammatory cells infiltration in the duodenum and AUC_5_ value in ECP patients. *C*. The relationship between the severity of inflammatory cells infiltration in the duodenum and AUC_5_ value in RFD-P patients. *D*. The relationship between the severity of inflammatory cells infiltration in the duodenum and AUC_15_ value in ECP patients *E*. The relationship between the severity of inflammatory cells infiltration in the duodenum and AUC_15_ value in RFD-P patients.

To investigate whether duodenal inflammation affects the early phase of gastric emptying, we investigated the relationship between duodenal inflammatory cells infiltration and AUC_5_ or AUC_15_ values in ECP and RFD-P patients. There was no significant difference (1.74±0.17, 1.91±0.21; p = 0.53) in the severity of duodenal inflammatory cells infiltration between ECP and RFD patients (Fig 5*A*). Then, there was a negative, but not significant, correlation between severity of duodenal inflammatory cells infiltration and AUC_5_ or AUC_15_ values in ECP and RFD-P patients (Fig 5*B*–5*E*).

## Discussion

The major findings of this study are: 1) 24.5% of FD patients refractory to anti-acid therapy were determined as early chronic pancreatitis using endosonography. 2) Abdominal pain score in GSRS score in early chronic pancreatitis (ECP) patients was significantly (P = 0.04) lower compared to that in RFD-P patients; 3) The early phase of gastric emptying in ECP and RFD-P patients were significantly disturbed compared to that in RFD patients; 4)There were no significant differences in clinical symptoms associated with high dietary intake of fat among the three groups.

The current study is first to report that close to 25% of RFD patients evaluated by endosonography fit the diagnosis of ECP according to the new standards provided by the Japan Pancreatic Association and previous studies [13, 22]. Considering our data and previous studies, we would like to suggest a new clinical approach for the identification and treatment of ECP patients and to provide separate diagnostic criteria that distinguish between ECP and RFD-P patients to determine the precise diagnosis of these distinct diseases. An accurate diagnosis of ECP would help prevent the progress of ECP into chronic pancreatitis and pancreatic cancer. Interestingly, since in this study, nearly 50% (25/50) of RFD-P patients [22] were compatible for ECP patients, we think endoscopic ultrasonography (EUS) was a useful tool to distinguish ECP patients from RFD-P patients as described in Fig 1. Thus, patients with EUS features with scores >2 may benefit from timely identification and treatment of ECP; that is, patients who receive an accurate diagnosis of ECP can benefit from the supplementation of pancreatic enzymes and the treatment of chronic pancreatitis, and their disease could be prevented from progressing into pancreatic tumor [21, 36, 37]. Ashizawa et al have reported that camostat mesilate, a common treatment for pancreatitis, is a significantly more effective therapy than famotidine in the treatment of epigastralgia in FD patients [15]. Considering the facts that pancreatic exocrine insufficiency may cause steatorrhea, weight loss and malnutrition-related complications, and that pancreatic cancer is considered as one of the most lethal malignancies, it is critical to learn how to differentiate between FD and ECP to make a proper diagnosis. In addition, further studies will be needed to evaluate the cost-benefit of the proposed measures, taking into account the high prevalence of functional dyspepsia.

In this study, we aimed to determine whether the characteristics of ECP patients differ from those of RFD-P and RFD patients. Our data shows that, with the exception of gender, the characteristics of ECP patients were similar to those of patients with RFD-P and RFD, as described in Table 1. In addition, the reason why the female population was dominant in the patients with RFD-P was not determined. Of interest, in our data, scores for bothersome postprandial distress symptoms in ECP were similar to those in RFD-P patient, as described in Fig 2. Although abdominal pain is generally considered one of the critical criteria in the diagnosis of ECP, postprandial abdominal fullness and early satiety may be associated with the etiology of ECP patients. Further studies will be needed to determine which factors are associated with abdominal pain and/or postprandial symptoms in the patients with ECP.

Currently, since EUS has been proven a useful modality to identify subtle pancreatic abnormalities that may be missed by US and CT, the close proximity of the high-frequency transducer to the pancreas allows detection of slight pathological changes in the pancreatic ducts and parenchyma [38]. In particular, studies have shown that EUS has the sensitivity to discover slight pathological changes associated with chronic pancreatitis [38, 39]. However, there is a lack of endosonographic data available that associate pancreatic abnormalities with clinical symptoms, gastric motility and levels of the various pancreatic enzymes in patients with concomitant functional dyspepsia. We are first to report that certain clinical symptoms and gastric motility among three groups using endosonography. Sahai et al have reported that the mean number of endosonographic abnormalities was higher in dyspeptic patients than in control patients [12]. Since, in Japan, the evaluation of EUS characteristics of ECP excludes major A factors, such as hyperechoic foci with shadowing and MPD calcification in Rosemont criteria [40], scores of EUS features in ECP patients tended to be low. In our study, patients with scores of EUS features >5 could not be enrolled as cases of ECP. Recent studies have shown that the total number of EUS criteria correlates with the severity of pancreatographic changes and with reduction in secretin-stimulated duodenal bicarbonate [41, 42]. Therefore, further studies are warranted to determine the correlation between each EUS feature and pancreatic enzyme abnormalities, clinical symptoms and gastric motility in these patients.

In this study, we compared gastric motility among ECP, RFD-P and RFD patients and found no significant differences in gastric emptying including Tmax and T_1/2_ values among the three groups. There have been conflicting reports regarding gastric emptying in patients with pancreatic enzyme abnormalities [43–45]. Gastric emptying has been shown to be accelerated in patients with pancreatic insufficiency [43, 44] and delayed in those without insufficiency [45]. Interestingly, our study shows that the early phase of gastric emptying in the patients with ECP was significantly disturbed compared to that in the patients with RFD. Kusano et al [46] and our previous studies [34, 35] have shown that FD can be attributed to either rapid or delayed gastric emptying. Similarly, the early phase of gastric emptying in the patients with RFD-P was also significantly disturbed compared to that in the patients with RFD. In contrast, there were no significant differences in the early phase of gastric emptying between ECP patients and RFD-P patients. Considering of the evaluation for some RFD-P patients in the view of EUS score in the two years follow-up, EUS scores of three RFD-P patients are aggravated (from score 0 to score 1) and two RFD-P patients advanced into ECP patients (data not shown). Therefore, we think that the certain population of RFD-P patients may be very early phase of ECP. Further studies will be needed to determine how populations of RFD-P patients will advance into ECP patients in several years later.

Pilichiewicz et al have reported that inhibition of fat digestion by a lipase inhibitor, orlistat, induced rapid gastric emptying [47]. Considering our data and previous reports, disturbance of digestion of fat in patients with pancreatic enzyme abnormalities may be associated with the rapid early phase of gastric emptying. Although, in our study, there were not significant differences in the gastrointestinal symptoms associated with the fat intake among the three groups, we have yet to apply the PFD (pancreatic function diagnostant) test to evaluate the actual grade of pancreatic dysfunction in the patients with RFD-P and ECP. Feinle-Bisset and Azpiroz et al [48] have shown that the manner in which fat is absorbed depends on the acyl chain length, which may explain in part the fact that fat droplet size also affects gut function and clinical symptoms. Considering this report, it appears that proper understanding of the relationship between fat intake and various GI symptoms would require analysis of the number of carbons present in the fatty acids consumed during meals. Considering that duodenal inflammation has been reported to be linked with FD patients [49–51] and GLP-1 production inhibited gastric emptying [52, 53], in our data, duodenal inflammation may be negatively associated with the early phase of gastric emptying such as AUC_5_ and AUC_15_ values through the upregulation of GLP-1 production induced by duodenal inflammation. Further studies will be warranted to investigate whether supplementation of a particular pancreatic enzyme and reduction of duodenal inflammation can improve the disturbance of the early phase of gastric emptying.

This study was carried out as a prospective study in a single center with a limited number of patients. Therefore, a prospective, randomized, multicenter study is required to confirm these findings. Another limitation is the evaluation of clinical symptoms for fat intake in this study. Since in this study, the evaluation of clinical symptoms for fat intake was based on the subjects own perception of how they respond to meals, further studies will be needed to determine their evaluations when the investigators feed the examined meals to the patients. Within the limitations of this study, there was no significant difference in severity of epigastric pain and postprandial abdominal fullness among ECP, RFD-P and RFD patients. Evaluation of the grade of abdominal pain and early phase of gastric emptying will be a useful tool to distinguish ECP patients from RFD-P patients or RFD patients, respectively. Further studies will be warranted to determine how clinical symptoms and impairment of the early phase of gastric emptying associate with the advance of ECP.
